# MASK‐air^®^ real‐world data in respiratory allergy in old‐age adults

**DOI:** 10.1002/clt2.12216

**Published:** 2023-01-16

**Authors:** Luis Taborda‐Barata, Maria Teresa Ventura, Hubert Blain, Luisa Brussino, Violeta Kvedariene, Désirée E. Larenas‐Linneman, Nhân Pham‐Thi, Boleslaw Samolinski, Joao A. Fonseca, Jean Bousquet, Bernardo Sousa‐Pinto

**Affiliations:** ^1^ UBIAir ‐ Clinical & Experimental Lung Centre University of Beira Interior Covilhã Portugal; ^2^ CICS‐UBI Health Sciences Research Centre University of Beira Interior Covilhã Portugal; ^3^ Department of Immunoallergology Cova da Beira University Hospital Centre Covilhã Portugal; ^4^ Unit of Geriatric Immunoallergology University of Bari Medical School Bari Italy; ^5^ Department of Geriatrics Montpellier University Hospital MUSE Montpellier France; ^6^ Department of Medical Sciences, Allergy and Clinical Immunology Unit University of Torino & Mauriziano Hospital Torino Italy; ^7^ Institute of Biomedical Sciences Department of Pathology Faculty of Medicine Vilnius University and Institute of Clinical Medicine Vilnius Lithuania; ^8^ Clinic of Chest Diseases and Allergology Faculty of Medicine Vilnius University Vilnius Lithuania; ^9^ Center of Excellence in Asthma and Allergy Médica Sur Clinical Foundation and Hospital México City Mexico; ^10^ Ecole Polytechnique Palaiseau, IRBA (Institut de Recherche Bio‐Médicale des Armées) Bretigny France; ^11^ Department of Prevention of Environmental Hazards, Allergology and Immunology Medical University of Warsaw Warsaw Poland; ^12^ MEDCIDS ‐ Department of Community Medicine, Information and Health Decision Sciences Faculty of Medicine, University of Porto Porto Portugal; ^13^ CINTESIS@RISE ‐ Health Research Network MEDCIDS Faculty of Medicine University of Porto Porto Portugal; ^14^ Charité – Universitätsmedizin Berlin Corporate Member of Freie Universität Berlin and Humboldt‐Universität zu Berlin Berlin Germany; ^15^ Fraunhofer Institute for Translational Medicine and Pharmacology ITMP Allergology and Immunology Berlin Germany; ^16^ University Hospital Montpellier Montpellier France

To the editor,

1

Real‐world data obtained by the MASK‐air^®^ (Mobile Airways Sentinel networK for airway diseases) app have had an impact on the knowledge about the phenotypes and management of respiratory allergic diseases.^1^ Studies assessing MASK‐air^®^ data have traditionally included users ranging in age from 16 to over 90 years, and a recent paper has shown that elderly users (≥65 years) can use the MASK‐air^®^ app after a short training period.^2^ However, it is not known whether the characteristics of elderly users differ from those of younger users.

The MASK‐air^®^ app is a DG Santé Good Practice for digitally‐enabled, patient‐centred care in rhinitis and asthma multi‐morbidity.^3^ In MASK‐air^®^, users are requested to daily report their global allergy, nose, eye and asthma symptoms, through visual analogue scales (VASs; scale of 0–100). Users are also requested to report their daily allergy medication use.

In this study, we compared users <65 and ≥65 years, namely regarding their demographic characteristics, reported allergy symptoms and reported medication use. Elderly patients are commonly studied as a single group of adults ≥65 years of age.^4^ However, a sub‐classification has been proposed: 65–74, 75–84 and ≥ 85 years. This may more adequately reflect pathophysiological changes in older people since, after 75 years of age, frailty and cognitive impairment become more common.^5^ In this paper, we compared users between 65 and 74 years with those ≥75 years, as there were too few observations from users ≥85 years.

We included all MASK‐air^®^ users ≥16 years of age from May 2015 to May 2022. Those from Italy were excluded since participants from Puglia had received training on how to use MASK‐air^®^.^2^

For comparison of different age groups, effect size measures for differences in proportions and medians were estimated. Effect size measures <0.2 indicate non‐meaningful differences, between 0.2 and 0.5 small differences, between 0.5 and 0.8 moderate differences and higher than 0.8 large differences.^6^

We assessed 19,369 users <65 years (333,395 days) and 519 users ≥65 (15,650 days) from 24 countries (Table 1). Among the users ≥65 years, most were <75 (*N* = 455; *N* days = 15,038). Each user <65 years reported an average of 17 days in MASK‐air^®^ versus 30 days for users ≥65 years.

**TABLE 1:** Frequency of days by country

	Days from patients aged <65 years—*N* (%)	Days from patients aged ≥65 years—*N* (%)
Argentina	5040 (1.5)	183 (1.2)
Australia	2555 (0.8)	42 (0.3)
Austria	7506 (2.3)	133 (0.8)
Belgium	1879 (0.6)	176 (1.1)
Brazil	11,776 (3.5)	128 (0.8)
Canada	476 (0.1)	3 (0.02)
Czech Republic	1751 (0.5)	460 (2.9)
Denmark	1343 (0.4)	0
Finland	5981 (1.8)	13 (0.1)
France	20,596 (6.2)	1690 (10.8)
Germany	30,747 (9.2)	2581 (16.5)
Great Britain	6833 (2.0)	822 (5.3)
Greece	9402 (2.8)	269 (1.7)
Hungary	708 (0.2)	8 (0.1)
Japan	4660 (1.4)	377 (2.4)
Lebanon	651 (0.2)	0
Lithuania	50,786 (15.2)	958 (6.1)
Mexico	73,531 (22.1)	4315 (27.6)
The Netherlands	8889 (2.7)	949 (6.1)
Poland	23,059 (6.9)	560 (3.6)
Portugal	18,347 (5.5)	1265 (8.1)
Slovenia	1362 (0.4)	324 (2.1)
Spain	28,229 (8.5)	103 (0.7)
Sweden	1754 (0.5)	204 (1.3)
Switzerland	5902 (1.8)	75 (0.5)
Turkey	9632 (2.9)	12 (0.1)

Days of users <65 and ≥65 years had overall similar clinical characteristics and asthma and rhinitis medication patterns (Table 2A). For daily reported symptoms, differences in VAS global allergy symptoms and VAS nose were not meaningful. However, meaningful differences were observed for VAS eye (effect size = 0.36) and VAS asthma (effect size = 0.95), whose median values were higher for elderly patients than for younger ones.

**TABLE 2:** Characteristics and outcomes of the days from assessed MASK‐air^®^ users with self‐reported rhinitis according to the age group

A. Comparison of days from patients under versus above 65 years			
	Days from patients aged <65 years (*N* = 333,395)	Days from patients aged ≥65 years (*N* = 15,650)	Effect size^a^
*N* users (average days per user)	19,369 (17.2)	519 (30.2)	‐
MASK‐air^®^ adherence (%)—median (IQR)	0.3 (1.3)	0.4 (1.6)	‐
Females—*N* (%)	192,513 (57.7)	5154 (32.9)	0.50
Age—mean (SD)	36.5 (12.6)	68.4 (3.0)	‐
VAS global allergy symptoms—median (IQR)	12 (27)	13 (20)	0.06
VAS nose—median (IQR)	12 (28)	13 (21)	0.06
VAS eyes—median (IQR)	4 (17)	7 (22)	0.36
VAS asthma—median (IQR)			
All users	0 (10)^b^	1 (11)^b^	0.95
Users with reported asthma	7 (22)	14 (37)	0.48
Allergic rhinitis CSMS^b^—median (IQR)	10 (18)	11 (16)	0.06
Total days reporting rhinitis medication—*N* (%)	156,311 (46.9)	7193 (46.0)	0.02
Oral antihistamines monotherapy	57,097 (17.1)	2031 (13.0)	0.12
Intranasal steroids monotherapy	31,901 (9.6)	1539 (9.8)	0.01
Azelastine‐fluticasone monotherapy	12,100 (3.6)	1042 (6.7)	0.14
Oral antihistamines + intranasal steroids	31,092 (9.3)	1346 (8.6)	0.03
Azelastine‐fluticasone + other rhinitis medication	11,415 (3.4)	478 (3.1)	0.02
Allergen immunotherapy^c^—*N* (%)	103,792 (31.1)	4593 (29.3)	0.04
Self‐reported asthma—*N* (%)	126,201 (37.9)	5490 (35.1)	0.06
Total days reporting asthma medication—*N* (%)	68,313 (20.5)	3938 (25.2)	0.11
SABA	8647 (2.6)	408 (2.6)	0
ICS	25,738 (7.7)	1484 (9.5)	0.06
ICS + LABA	37,457 (11.2)	2806 (17.9)	0.19
LAMA or biologics	2387 (0.7)	40 (0.3)	0.06
Other medications	14,979 (4.5)	829 (5.3)	0.04
Conjunctivitis—*N* (%)	240,481 (72.1)	10,347 (66.1)	0.13
Baseline symptoms^d^—median (IQR)	5 (3)	4 (4)	0.34
Baseline impact^e^—median (IQR)	1 (3)	1 (3)	0
B. Comparison of days from patients aged 65–74 years versus over 75 years			
	Days from patients aged 65–74 years (*N* = 15,038)	Days from patients aged ≥75 years (*N* = 612)	Effect size^a^
*N* users (average days per user)	455 (33.1)	70 (8.7)	‐
MASK‐air^®^ adherence (%)—median (IQR)	0.4 (1.6)	0.2 (0.9)	‐
Females—*N* (%)	4991 (33.2)	163 (26.6)	0.14
Age—mean (SD)	68.0 (2.4)	76.8 (2.2)	‐
VAS global allergy symptoms—median (IQR)	13 (20)	16 (25)	0.23
VAS nose—median (IQR)	13 (21)	15 (23)	0.15
VAS eyes—median (IQR)	7 (22)	13 (20)	0.47
VAS asthma—median (IQR)			
All users	1 (10)^c^	9 (22)^c^	0.84
Users with reported asthma	14 (37)	11 (39)	0.18
Allergic rhinitis CSMS^b^—median (IQR)	10 (16)	15 (21)	0.42
Total days reporting rhinitis medication—*N* (%)	6752 (44.9)	441 (72.1)	0.56
Oral antihistamines monotherapy	1867 (12.4)	164 (26.8)	0.37
Intranasal steroids monotherapy	1519 (10.1)	20 (3.3)	0.28
Azelastine‐fluticasone monotherapy	943 (6.3)	99 (16.2)	0.32
Oral antihistamines + intranasal steroids	1308 (8.7)	38 (6.2)	0.10
Azelastine‐fluticasone + other rhinitis medication	395 (2.6)	83 (13.6)	0.43
Allergen immunotherapy^c^—*N* (%)	4593 (30.5)	0 (0)	1.17
Self‐reported asthma—*N* (%)	5120 (34.0)	370 (60.5)	0.54
Total days reporting asthma medication—*N* (%)	3775 (25.1)	163 (26.6)	0.03
SABA	345 (2.3)	63 (10.3)	0.35
ICS	1429 (9.5)	55 (9.0)	0.02
ICS + LABA	2709 (18.0)	97 (15.9)	0.06
LAMA or biologics	37 (0.2)	3 (0.5)	0.05
Other medications	766 (5.1)	63 (10.3)	0.20
Conjunctivitis—*N* (%)	9894 (65.8)	453 (74.0)	0.18
Baseline symptoms^d^—median (IQR)	4 (4)	4 (4)	0
Baseline impact^e^—median (IQR)	1 (3)	1 (2)	0

Abbreviations: CSMS, Combined symptom‐medication score; ICS, Inhaled corticosteroids; IQR, Interquartile range; LABA, Long‐acting beta‐agonists; LAMA, Long‐acting muscarinic antagonists; SABA, Short‐acting beta‐agonists; SD, Standard‐deviation; VAS, Visual analogue scale.

^a^ Effect size measures <0.2 indicate non‐meaningful differences, between 0.2 and 0.5 indicate small differences, between 0.5 and 0.8 indicate moderate differences, and higher than 0.8 indicate large differences.

^b^ The CSMS ranges from 0 to 100. Its formula is [(0.037 × VAS global symptoms) + (0.033 × VAS eyes) + (0.020 × VAS nose) + (0.027 × VAS asthma) + (0.450 if azelastine‐fluticasone is used) + (0.424 if nasal steroids are used) + (0.243 if asthma medication is used) + (0.380 if other rhinitis relief medication is used)] × 7.577). Its description can be found in Sousa‐Pinto et al. Allergy. 2022; 77(7):2147–2162^7^.

^c^ Includes subcutaneous and sublingual immunotherapy, but not treatment on biologics.

^d^ Number of allergic rhinitis symptoms reported by the user (including runny nose, itchy nose, sneezing, congestion, impaired smell, red eyes, itchy eyes and watery eyes).

^e^ Number of domains affected by allergy symptoms reported by the user (including sleep, daily activities, participation in school or work and overall activities).

Comparing days from users 65–74 years of age with those ≥75 (Table 2B), we observed small and moderate effect size measures in some clinical, medication and symptom‐related variables. Meaningful differences were observed for VAS eye (effect size = 0.47), VAS asthma (effect size = 0.84) and the combined symptom‐medication score (effect size = 0.42), with higher median values being observed in older patients.

We observed that levels of patients' reported outcomes tend to increase with age, in particular median VAS asthma and, to a lesser extent, VAS eyes. Meaningful differences were rarely observed when comparing days from users <65 and ≥65 years, suggesting similar clinical and medication use patterns. By contrast, larger differences were observed when considering users 65–74 versus ≥75 years, with the latter reporting not only more severe symptoms but also more days of rhinitis treatment and different treatment patterns. The average number of reported days was higher for patients ≥65 years than for those <65 years, hinting at the usability and acceptability of MASK‐air^®^ among the former. One of the major limitations of this study is the low number of users ≥75 years. There were two countries for which there were no patients ≥65 years (Denmark and Lebanon), but this will probably not have had a relevant impact on our results, as users from these two countries combined provided only 0.6% of all reported days in MASK‐air^®^.

This study suggests that MASK‐air^®^ studies may include patients of up to at least 75 years. However, this issue should be addressed in a larger sample of patients in this age range.

## AUTHOR CONTRIBUTIONS

Luis Taborda‐Barata proposed the study and participated in the data collection; Maria Teresa Ventura and Hubert Blain co‐proposed the study and participated in the analysis; Jean Bousquet participated in the conceptualisation, formal analysis, supervision and writing ‐ original draft. Bernardo Sousa‐Pinto participated in the methodology, formal analysis and writing ‐ original draft. Joao A. Fonseca participated in the conceptualisation, formal analysis, supervision and writing ‐ review & editing. All remaining authors participated in the data collection and writing ‐ review & editing.

## CONFLICTS OF INTEREST

Jean Bousquet reports personal fees from Chiesi, Cipla, Hikma, Menarini, Mundipharma, Mylan, Novartis, Sanofi‐Aventis, Takeda, Teva, Uriach, other from KYomed‐Innov and MASK‐air, personal fees from Purina. There were no COIs for the other authors.
